# Impact of the Pandemic on the Diagnosis and Treatment of Patients With Urological Tumors at a University Hospital

**DOI:** 10.7759/cureus.21357

**Published:** 2022-01-18

**Authors:** Fernando Nestor Facio, Ana Clara Nagle Spessoto, Maria Fernanda Warick Facio, Luís Cesar Fava Spessoto

**Affiliations:** 1 Urology, Faculty of Medicine of São José do Rio Preto (FAMERP), São José do Rio Preto, BRA; 2 Internal Medicine, Medical School of Catanduva, Catanduva, BRA; 3 Medicine, Faceres Medical School, São José do Rio Preto, BRA

**Keywords:** urology, coronavirus, therapy, diagnosis, cancer

## Abstract

Background

The rapid dissemination of the coronavirus 2019 (COVID-19) had dramatic effects on individuals and healthcare systems in 2020. At our tertiary hospital, surgeries were recommended for patients at high oncological risk, with the prioritization of the maintenance of care and treatment of cancer. We aimed to assess the impact of the pandemic on the diagnosis and treatment of patients with urological tumors under the care of a university hospital.

Materials and methods

A retrospective analysis was performed of the charts of patients with urological tumors (prostate, kidney, bladder, and testicle) under the care and treated surgically at the Hospital de Base in 2019 and 2020, independently of ethnicity. The results were compared by the chi-square test (with a significance level of 5%).

Results

A discrete increase occurred in the quantity of appointments in 2020 (n = 5,846) compared to 2019 (n = 5,726). The most frequent types of cancer in 2019 and 2020 were, respectively, prostate (70.97% and 73.37%), bladder (18.07% and 12.52%), kidney (7.96% and 8%), and testicle (29.24% and 70.76%). Analyzing 279 surgeries performed on patients with prostate tumors, a 12.7% increase occurred in the year of the pandemic. Analyzing 271 surgical procedures on patients with bladder cancer, no considerable change occurred.

Conclusion

The analysis of the impact of the pandemic on the diagnosis and treatment of patients with urological tumors at a university hospital revealed a discrete increase in the number of outpatient appointments and a slight reduction in the number of patients and surgical procedures in the year of the pandemic (2020). More surgical procedures were conducted on patients with prostate cancer in 2020. More patients with bladder cancer sought medical care in the year of the pandemic, and practically the same quantity was submitted for surgical treatment. There was no statistically significant difference among types of cancer.

## Introduction

The disease, denominated coronavirus 2019 (COVID-19) in Wuhan, China [1], spread quickly to other regions of the country and around the world [2,3]. This pandemic has had implications regarding the continuity of the treatment of patients, especially oncological patients. A change in priority occurred, with human, medical, and paramedical resources being directed at the care of patients infected with COVID-19. Moreover, measures to confine the population have led to restrictions on travel for appointments, examinations, and treatment (clinical and surgical).

Patients with cancer are considered immunocompromised due to the nature of the disease as well as the treatment to which they are submitted (chemotherapy, radiotherapy, or surgery). Considering the more advanced age, the impossibility of receiving adequate medical care, and the fact that patients with cancer have a 3.5-fold greater risk of developing severe events related to COVID-19, all elective surgeries, chemotherapy, and radiotherapy procedures in stable patients should be postponed [4-6]. The decision to postpone clinical appointments or oncological surgeries currently depends on the capacity of the hospital in terms of infrastructure and the support necessary for these types of procedures [7].

In this context, the majority of urological medical centers must prioritize surgical procedures for oncological patients, applying restrictions to elective non-oncological procedures with the aim of optimizing health resources and minimizing the risk of hospital infection [8]. At our tertiary hospital, surgeries were recommended for patients at high oncological risk, prioritizing the maintenance of care and treatment for these patients.

Considering the changes to the care of oncological patients caused by the pandemic, an investigation is warranted into the impact on the diagnosis and treatment of patients with urological tumors at the second-largest hospital complex in the state of São Paulo, Brazil. Theoretically, a reduction would be expected in the quantity of appointments and surgical procedures for these patients. Therefore, the aim of the present study was to assess the impact of the pandemic on the diagnosis and treatment of patients with urological tumors at a university hospital in terms of the number of outpatient appointments with a diagnosis of urological tumors and the quantity of surgical procedures performed on patients with urological tumors.

## Materials and methods

A retrospective, descriptive, cross-sectional study was conducted involving the analysis of the charts of patients with urological tumors (prostate, bladder, kidney, and testicle) at the Hospital de Base, São José do Rio Preto, SP, Brazil, independently of ethnicity. The period study was 2019 and 2020 (pandemic). This study received approval from the institutional review board (certificate number: 44639621.0.0000.5415).

The inclusion criterion was a diagnosis of urological tumors in 2019 and 2020 (pandemic) in pediatric and adult patients who may have undergone surgical treatment in the period. The exclusion criterion was a diagnosis in the period prior to 2019.

An analysis was conducted on the quantity of outpatient appointments for patients with a diagnosis of urological tumors, the quantity of surgical procedures performed on these patients, and the possible association between the patients diagnosed and the respective surgical treatment. All the data used in this study were from the institutional computational system.

Data analysis involved the calculation of relative frequencies using Microsoft Office Excel® (Microsoft Corporation, Redmond, Washington, USA). The results were compared by the chi-square test (with a significance level of 5%).

## Results

A discrete increase occurred in the quantity of appointments in 2020 (n = 5,846) compared to 2019 (n = 5,726). The most frequent types of cancer in 2019 and 2020 were, respectively, prostate (70.97% and 73.37%), bladder (18.07% and 12.52%), kidney (7.96% and 8%), and testicle (29.24% and 70.76%) (Table 1) (p > 0.05).

**Table 1:** Quantity of outpatient appointments for patients with a diagnosis of urological tumors at a university hospital.

Type	2019 N (%)	2020 N (%)
Prostate	4,064 (71.0)	4,289 (73.4)
Bladder	1,035 (18.1)	732 (12.5)
Kidney	456 (8.0)	468 (8.0)
Testicle	69 (1.2)	167 (2.9)
Total	5,726	5,846

A discrete reduction, not significant, occurred in the quantity of patients with a diagnosis of urological tumors at the university hospital in 2020 (n = 421) compared to 2019 (n = 447). In the year of the pandemic, a reduction occurred in the number of patients with prostate, kidney, and testicular tumors, whereas an increase occurred in the number of patients with bladder cancer (Table 2) (p > 0.05).

**Table 2:** Quantity of patients with a diagnosis of urological tumors at a university hospital.

Type	2019 N (%)	2020 N (%)
Prostate	241 (53.9)	196 (46.6)
Bladder	95 (21.3)	127 (30.2)
Kidney	95 (21.3)	84 (20.0)
Testicle	16 (3.6)	14 (3.3)
Total	447	421

Table 3 displays the quantity of surgical procedures performed on patients with a diagnosis of urological tumors at the university hospital. Analyzing 279 surgeries for patients with prostate tumors in the period of interest, 130 (46.6%) were performed in 2019 and 149 (53.4%) were performed in 2020, corresponding to a 12.7% increase in the year of the pandemic. Analyzing the 271 surgical procedures for patients with bladder cancer, 136 (50.2%) endoscopic resections were performed in 2019 and 135 (49.8%) were performed in 2020, constituting no considerable difference. Regarding the 157 surgical procedures for kidney tumors, 96 (61.1%) were performed in 2019 and 61 (38.9%) were performed in 2020, corresponding to a 38.9% reduction in the year of the pandemic. Among the 67 orchiectomies on patients with testicular tumors, 38 (56.7%) were performed in 2019 and 29 (43.3%) were performed in 2020, corresponding to a 23.7% reduction in this surgical procedure. There was no statistically significant difference between the groups.

**Table 3:** Quantity of surgical procedures of patients with a diagnosis of urological tumors at a university hospital.

Type	2019 N (%)	2020 N (%)
Prostate	130 (32.5)	149 (39.8)
Bladder	136 (34.0)	135 (36.1)
Kidney	96 (24.0)	61 (16.3)
Testicle	38 (9.5)	29 (7.8)
Total	400	374

The quantity of patients with a diagnosis of urological tumors and surgical procedures on these patients is expressed in Table 4.

**Table 4:** Quantity of patients with a diagnosis of urological tumors and surgical procedures on these patients at a university hospital.

Type	Patients		Procedures	
	2019	2020	2019	2020
Prostate	241	196	130	149
Bladder	95	127	136	135
Kidney	95	84	96	61
Testicle	16	14	38	29

## Discussion

The analysis of the impact of the pandemic on the diagnosis and treatment of patients with urological tumors at a university hospital revealed a discrete increase in the quantity of outpatient appointments and a slight reduction in the quantity of patients and surgical procedures in the year of the pandemic (2020). Regarding the organs affected, a reduction occurred in the quantity of patients with prostate cancer who sought treatment, but more surgical procedures were performed on these patients in 2020. More patients with bladder cancer sought treatment in the year of the pandemic, and practically the same quantity was submitted for surgical treatment. For kidney and testicular tumors, reductions occurred in both the demand for hospital care and the quantity of surgeries.

Exposure to possibly contaminated environments, such as medical centers, was expected to lead to a possible absence of patients during the current pandemic. However, only a slight decrease was found in the present study regarding the quantity of patients who sought hospital care for urological treatment and the number of surgical procedures performed. This may be related to the fact that care was not suspended in the period, as patients with urological tumors should receive care to ensure the earliest possible diagnosis and treatment.

A 12.7% increase occurred in the quantity of surgeries performed on patients with prostate tumors in the year of the pandemic. Early treatment in such cases is of the utmost importance, as 15,983 deaths due to prostate cancer occurred in Brazil in 2019 [9]. Moreover, there are no symptoms in the initial stages of this disease. Due to the reduction in the quantity of elective surgeries, oncological surgical interventions, including cases of prostate cancer, were considered a priority, which may explain the increase in the number of these procedures during the pandemic.

Analyzing patients with bladder cancer, no change occurred in the quantity of surgeries in the year of the pandemic. In such cases, patients have clinical conditions that hasten the prompt search for medical care, such as hematuria, clot formation, and urinary retention.

A reduction occurred in the quantity of surgical procedures on patients with kidney and testicular tumors during the pandemic. Patients with these tumors generally have few symptoms to justify the search for urgent medical care.

In times of crisis, uro-oncological surgeries should be concentrated at tertiary urology centers, which should ideally remain COVID-19-free environments to ensure high-complexity treatment for oncological patients. The adverse effects of the COVID-19 pandemic on urological health care will certainly include significant harm due to the postponement of oncological therapy, including surgeries [10].

Despite the pandemic, the authors believe that uro-oncological patients must have close follow-up because some neoplasias, such as prostate cancer, could disseminate metastatic with a poor prognosis.

Limitations

This work is based on a retrospective study performed at a single medical center. Moreover, urological tumors less frequently, such as penile tumors, were excluded. Therefore, long-term and multi-centric studies are necessary to confirm our results.

## Conclusions

In the present study, the analysis of the impact of the pandemic on the diagnosis and treatment of patients with urological tumors at a university hospital revealed a discrete increase in the quantity of outpatient appointments as well as a slight reduction in the quantity of patients and surgical procedures in the year of the pandemic (2020). More surgical procedures were conducted on patients with prostate cancer in 2020. More patients with bladder cancer sought medical care in the year of the pandemic, and practically the same quantity was submitted for surgical treatment.
